# Quantitative approaches for studying G protein-coupled receptor signalling and pharmacology

**DOI:** 10.1242/jcs.263434

**Published:** 2025-01-15

**Authors:** Abigail Pearce, Theo Redfern-Nichols, Edward Wills, Matthew Rosa, Iga Manulak, Claudia Sisk, Xianglin Huang, Peace Atakpa-Adaji, David L. Prole, Graham Ladds

**Affiliations:** Department of Pharmacology, University of Cambridge, Tennis Court Road, Cambridge, CB2 1PD, UK

**Keywords:** GPCRs, cAMP, IP_3_, Ca^2+^ signalling, Cell signalling, BRET, FRET

## Abstract

G protein-coupled receptor (GPCR) signalling pathways underlie numerous physiological processes, are implicated in many diseases and are major targets for therapeutics. There are more than 800 GPCRs, which together transduce a vast array of extracellular stimuli into a variety of intracellular signals via heterotrimeric G protein activation and multiple downstream effectors. A key challenge in cell biology research and the pharmaceutical industry is developing tools that enable the quantitative investigation of GPCR signalling pathways to gain mechanistic insights into the varied cellular functions and pharmacology of GPCRs. Recent progress in this area has been rapid and extensive. In this Review, we provide a critical overview of these new, state-of-the-art approaches to investigate GPCR signalling pathways. These include novel sensors, Förster or bioluminescence resonance energy transfer assays, libraries of tagged G proteins and transcriptional reporters. These approaches enable improved quantitative studies of different stages of GPCR signalling, including GPCR activation, G protein activation, second messenger (cAMP and Ca^2+^) signalling, β-arrestin recruitment and the internalisation and intracellular trafficking of GPCRs.

## Teaser

**Summary:** We provide a critical overview of approaches used to quantitatively study G protein-coupled receptor signalling in cells.

## Introduction

**Fig. 1.**
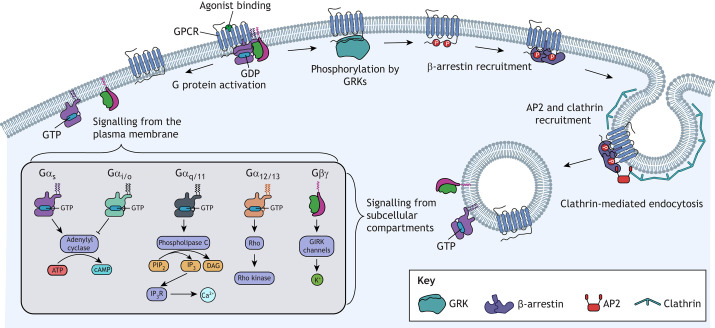
**Overview of GPCR signalling.** Following agonist binding, the GPCR undergoes a conformational change, enabling it to bind and activate a heterotrimeric G protein. G protein signalling is determined primarily by the type of Gα subunit, which falls into one of four subfamilies: Gα_s_, which is coupled to activation of adenylyl cyclase and production of cAMP; Gα_i/o_, which is coupled to inhibition of adenylyl cyclase and activation of phosphodiesterase to decrease cAMP; Gα_q/11_, which is coupled to activation of phospholipase C and subsequent mobilisation of intracellular Ca^2+^ through IP_3_ receptors; and Gα_12/13_, which is coupled to activation of Rho GTPases. The Gβγ complex can also mediate signalling by, for example, activating G protein-coupled inwardly rectifying potassium (GIRK) channels. Activated GPCRs are phosphorylated by GRKs, enabling recruitment of β-arrestins. β-arrestins can interact with the AP2 complex, which then interacts with clathrin. Recruitment of clathrin elicits the formation of a clathrin-coated pit, followed by endocytosis and receptor internalisation. Following internalisation, some GPCRs can continue signalling via G proteins from subcellular compartments. DAG, diacylglycerol; IP_3_R, IP_3_ receptor; Rho kinase, Rho-associated protein kinases. Created in BioRender by Redfern-Nichols, T., 2025. https://BioRender.com/w94r168. This figure was sublicensed under CC-BY 4.0 terms.

G protein-coupled receptor (GPCR) signalling pathways underlie numerous physiological processes such as cardiovascular function, neurotransmission, taste, smell, vision and metabolism. These pathways are implicated in many human diseases and are the targets of ∼30% of clinically used drugs (Hauser et al., 2017). There are more than 800 GPCRs, which transduce a vast array of extracellular stimuli into a variety of intracellular signals via activation of associated heterotrimeric G proteins (which are composed of Gα, Gβ and Gγ subunits) and multiple downstream effectors (Fig. 1).

Binding of an agonist to a GPCR causes conformational changes that trigger exchange of guanosine triphosphate (GTP) for guanosine diphosphate (GDP) on the Gα subunit, dissociation of activated Gα from the Gβ–Gγ dimer (Gβγ), and dissociation of Gα and Gβγ from the GPCR. The Gα and Gβγ subunits can then initiate downstream signalling cascades by binding to a variety of effector proteins. Heterotrimeric G protein complexes are diverse, with Gα subunits being classified into four major families: Gα_s_, Gα_i/o_, Gα_q/11_ or Gα_12/13_ (Fig. 1) (Syrovatkina et al., 2016; Wootten et al., 2018). A variety of Gβγ dimer subunits expands the diversity further. Different heterotrimeric G protein complexes can couple to distinct signalling pathways, thereby transducing changes in GPCR activity into different intracellular signals. Therefore, each GPCR has the potential to evoke multiple different intracellular second messenger signals, such as Ca^2+^ release or production or inhibition of cyclic adenosine 3′,5′ monophosphate (cAMP), by coupling to different G proteins and other signalling mediators (Wootten et al., 2018). These differential responses can be ‘biased’ by factors such as the agonist used or by protein modulators (Harris et al., 2021 preprint; Wootten et al., 2018), which can selectively affect the coupling of GPCRs to specific G proteins. This bias has important consequences for cellular physiology and pharmacology (Wootten et al., 2018). Following activation, some GPCRs can be phosphorylated by GPCR kinases (GRKs), enabling recruitment of β-arrestins, which can uncouple the G proteins. In addition, agonist-occupied GPCRs can undergo receptor internalisation, after which they can continue to signal from intracellular compartments, or undergo degradation or recycling to the plasma membrane.

This Review addresses the quantitative approaches that can be used to study GPCR signalling, encompassing the entire pathway from ligand binding to downstream signalling, β-arrestin recruitment and receptor internalisation. We provide a critical overview of the new and state-of-the-art approaches that comprise a growing experimental toolkit to expand the understanding of GPCR signalling.

## Fluorescence, luminescence and proximity assays for studying GPCR signalling

**Fig. 2.**
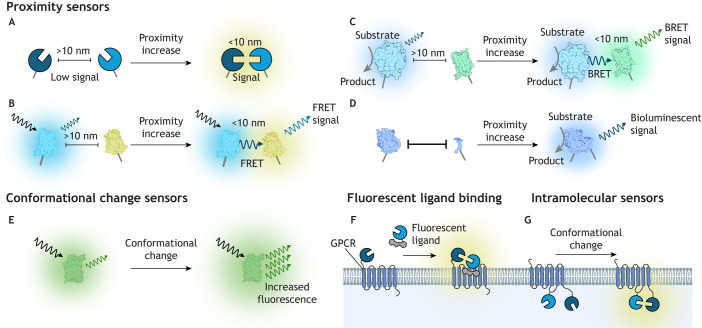
**Fluorescence and luminescence in assays for GPCR signalling.** Both luminescence and fluorescence measurements are versatile and common ways to assess GPCR signalling. Proximity sensors allow detection of appropriately oriented proteins in proximity (less than 10 nm, illustrated in A) (Kobayashi et al., 2019). These sensors often utilise (B) FRET or (C) BRET, which rely on the excitation of a fluorescent acceptor by a fluorescent or luminescent donor, respectively. (D) Another type of proximity assay uses luciferases or fluorescent proteins that are ‘split’ into two protomers, which reconstitute an intact protein when they approach close enough to bind (Bae et al., 2024). (E) Single-wavelength sensors that produce increased fluorescence or bioluminescence following a conformational change can also detect GPCR signalling events. Proximity assays can quantify upstream GPCR dynamics such as (F) ligand binding or (G) GPCR conformational changes through intramolecular proximity sensors. Created in BioRender by Redfern-Nichols, T., 2025. https://BioRender.com/y05d181. This figure was sublicensed under CC-BY 4.0 terms.

GPCR signalling can be measured in endogenous conditions or in perturbed systems (Box 1), and these measurements can provide insights into the kinetics of signalling or a predetermined endpoint (Box 2). Many recently developed assays involve fluorescent or bioluminescent components (Fig. 2). Bioluminescence relies on catabolism of a substrate by a luciferase enzyme and does not require an external light source, thereby minimising phototoxicity and autofluorescence; however, substrate depletion can affect signals. The relatively low light output of bioluminescence may restrict its applications to population assays, although newer luciferases such as the nanoluciferase NanoLuc (NLuc) offer greater light output and enable single-cell imaging (Kobayashi et al., 2019). In comparison, fluorescence produces relatively brighter output, enabling single-cell observations, but requires illumination that can generate autofluorescence, phototoxicity and photobleaching that constrains its use for time-lapse studies.

**Box 1. Use of endogenous versus perturbed systems for studying GPCR signalling**

Many of the techniques used to measure GPCR signalling rely on overexpression of the GPCR or signalling proteins, which are also often highly modified (e.g. the mini-G proteins; Wan et al., 2018) or tagged with fluorescent or bioluminescent proteins (e.g. the heterotrimeric G protein dissociation-based sensors; Olsen et al., 2020). These approaches enable the detection of specific protein–protein interactions, often in real time, which provides kinetic information (see box figure). However, overexpression of signalling proteins can perturb native signalling and obscure ligand bias (Wootten et al., 2018). Fusing bulky protein tags to signalling proteins can also alter their function, potentially influencing processes such as G protein coupling (Wright et al., 2024), protein–protein interactions, expression and compartmentalisation. Sensors that do not rely on overexpression or tagging may circumvent these issues. However, these methods are often specific to a family of effector proteins, rather than specific individual subtypes. Instead, the use of partially perturbed systems, where limited signalling elements are tagged, could enable reduced interference from overexpression or bulky fluorophores. For example, in translocation assays used to measure G protein signalling, such as EMTA, proximity between a tagged G protein effector and a plasma membrane-bound fluorophore is detected. There is increasing evidence that the G protein-coupling profile detected can depend on the sensor employed (Wright et al., 2024).
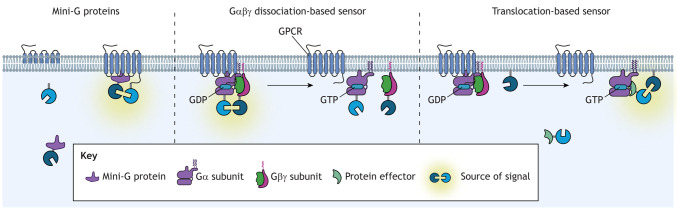

Created in BioRender by Redfern-Nichols, T., 2025. https://BioRender.com/d56s186. This figure was sublicensed under CC-BY 4.0 terms.

**Box 2. Fluorescent and luminescent sensors that enable kinetic versus endpoint measurements of GPCR signalling**

Proximity-based biosensors enable real-time detection of G protein activation as well as the recruitment and activation of downstream effector proteins. These assays yield valuable kinetic information but often rely on overexpression and/or tagged proteins, which can perturb endogenous signalling (Box 1). In addition to measuring protein–protein interactions, modified signalling proteins can also be used to directly measure second messengers such as cAMP and Ca^2+^ in live cells (see box figure), such as in the G-FLAMP, CUTie, GloSensor (e.g. pGloSensor-22f), CAMYEL, GCaMP and cameleon sensors. Though these likely have a reduced impact on GPCR signalling or agonist bias, they might still alter cellular processes, complicating interpretation of the results. In contrast, endpoint detection assays, such as the TR-FRET and HTRF cAMP kits available from Revvity (Preti et al., 2022), can measure GPCR signalling in primary tissues and unmodified cell lines, with cell lysis enabling the use of antibodies. Although endpoint assays often lack specificity for specific subtypes of GPCRs and G proteins, they are useful to confirm that signalling phenomena occur in a physiological setting. In many cases, application of both methodologies is required to fully elucidate the GPCR signalling cascade in a physiological setting.
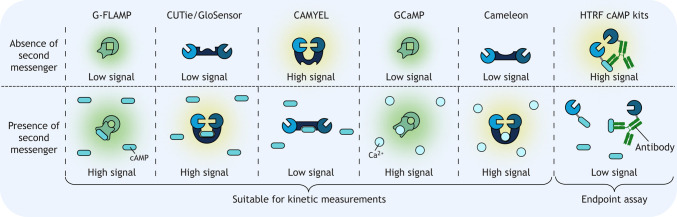

Created in BioRender by Redfern-Nichols, T., 2025. https://BioRender.com/b93r457. This figure was sublicensed under CC-BY 4.0 terms.

Proximity assays that measure luminescence and/or fluorescence output can provide quantification of protein–protein interactions and can also report conformational changes in GPCRs or downstream effectors (Fig. 2A). Bioluminescence resonance energy transfer (BRET) and Förster resonance energy transfer (FRET) are proximity assays that rely on excitation of a fluorescent acceptor by a luminescent or fluorescent donor, respectively (Fig. 2B,C). The efficiency of BRET and FRET is inversely proportional to the sixth power of the separation distance. Therefore, BRET or FRET can only occur when the two proteins of interest are in proximity (less than 10 nm), with appropriate orientations (Kobayashi et al., 2019), and when their excitation and emission spectra overlap appropriately. Another type of proximity assay relies on luciferases or fluorescent proteins that are ‘split’ into two protomers that only reconstitute an intact functional protein when they bind to each other (Bae et al., 2024) (Fig. 2D). A disadvantage of this method is the finite affinity of the split protomers for their complementing partner, which can introduce artefactual interactions and background signals in cells. Another class of proximity biosensors, conformational change sensors, relies on changes in fluorescence under different conditions, such as binding of a target factor, leading to a conformational change of the fluorophore that affects its output (Fig. 2E).

## Quantification of agonist binding and receptor activation

As GPCRs are transmembrane receptors, a crucial step in GPCR function is ligand recognition. Ligand–receptor specificity allows GPCRs to recognise a myriad of stimuli whilst still maintaining tissue and subcellular selectivity. Radioligand binding assays use ligands labelled with radioisotopes that do not alter the binding affinity of the ligand to its receptor (Holdgate, 2017) and allow detection of ligand–receptor binding using scintillation counters. The process is laborious, with each data point requiring an independent filtration step to separate GPCR-bound radioligand from free radioligand in solution, which takes time and reagents to complete (Gherbi et al., 2020). Scintillation proximity assays (SPAs) offer reduced acquisition times and enable 384-well plate scalability, achieving higher throughput (Zwier et al., 2010). However, fluorescence or bioluminescence assays are often preferred to radioligand binding assays because they do not require radioisotopes, can be applied in live cells and can be used to measure binding kinetics. Fluorescence-based ligand binding assays rely on fluorophore-tagged antagonists or agonists (Fig. 2F). Fluorescence output can be measured directly, using methods such as fluorescence polarisation (Gagne et al., 2002), or indirectly using resonance energy transfer (RET). The latter method offers better signal-to-noise ratios and lower non-specific signals (Cottet et al., 2011). For example, the Tag-Lite system (Zhang and Xie, 2012; Zwier et al., 2010) uses time-resolved FRET (TR-FRET). In this system, the GPCR is tagged with an *O*^6^-alkylguanine DNA alkyltransferase enzyme (SNAP-tag or CLIP-tag) at its extracellular N terminus, which can be irreversibly labelled with a terbium cryptate fluorophore substrate (Lumi4-Tb) that reacts with ligands tagged with green or red acceptor fluorophores (Emami-Nemini et al., 2013; Zhang and Xie, 2012). Further improvements over FRET-based techniques are provided by BRET-based assays. Whereas FRET donor excitation requires a laser, BRET donor excitation utilises a luciferase enzyme (Fig. 2B,C). BRET assays therefore do not require an additional light source, bypassing limitations associated with FRET such as photobleaching and autofluorescence (Koterba and Rowan, 2006; Stoddart et al., 2015; Suchankova et al., 2021). Because both FRET and BRET experiments can be performed in live cells, receptor–ligand binding kinetics can be observed, allowing for the calculation of on and off rates (*k*_on_ and *k*_off_, respectively) (Preti et al., 2022). However, the availability of cell lines expressing tagged GPCRs and the limited availability of fluorescent ligands constrains their use.

Following agonist binding, the GPCR undergoes a conformational change, termed receptor activation, that enables association and activation of the G protein. Receptor activation alone, without consideration of the G protein, can be measured using intramolecular sensors (Fig. 2G). FRET sensors with the donor and acceptor fluorophores located within the same receptor – for example, using cyan fluorescent protein (CFP) and yellow fluorescent protein (YFP) – were initially employed to measure this stage of GPCR activation (Vilardaga et al., 2003). Alternatively, CFP fused at the intracellular C terminus of a GPCR has been used as a FRET partner with a fluorescein arsenical hairpin binder (FlAsH) (Hoffmann et al., 2005) bound to a FlAsH-binding peptide sequence inserted into the intracellular loop 3 (ICL3) of the GPCR. However, these systems are receptor specific and not suitable for high-throughput screening of many GPCRs. A generalised strategy for BRET sensing in GPCRs, shown to be suitable for different receptors, has been developed utilising a C-terminal NLuc donor together with a HaloTag acceptor inserted into ICL3 and labelled with the HaloTag NanoBRET 618 ligand (Schihada et al., 2018). Receptor activation can also be determined using intermolecular sensors, such as nanobodies that are selectively recruited to the active receptor. By tagging the nanobody and the receptor with fluorophores or luciferases, activation can be measured using RET (El Daibani and Che, 2021). However, nanobodies are also receptor specific and have thus far only been used for a small range of receptors.

A more generalisable system for detecting GPCR activation utilises mini-G proteins. Mini-G proteins are smaller versions of Gα subunits, consisting primarily of the thermostabilised Gα GTPase domain with a reduced N terminus that no longer contains membrane-anchoring domains (Carpenter and Tate, 2016; Wan et al., 2018). Mini-G proteins were developed to stabilise active GPCR conformations; when tagged with fluorescent proteins, they can be used to measure G protein recruitment to active GPCRs (Wan et al., 2018) (Box 1). Mini-G proteins do not bind to the membrane or to Gβγ subunits and do not contribute to downstream signals. However, the movement of mini-G proteins to the membrane likely differs from that of endogenous Gα subunits, and an absence of binding to Gβγ likely affects the kinetics of mini-G protein recruitment and dissociation from GPCRs, relative to endogenous Gα. Furthermore, mini-G proteins might block internalisation of GPCRs and disrupt intracellular signalling (Manchanda et al., 2024). These limitations constrain the utility of mini-G proteins as sensors, especially when determining the specificity of G protein coupling.

## Quantifying G protein activation

Activated GPCRs can bind to many different proteins as a first step in signal transduction (Harris et al., 2021 preprint; Wootten et al., 2018). Most notably, different activation states of GPCRs can lead to differential binding to diverse G proteins, thereby linking GPCRs to different signalling pathways (Fig. 1) (Syrovatkina et al., 2016; Wootten et al., 2018). Methods for identifying the specific G protein complexes bound to GPCRs and the dynamics of these associations are necessary to fully understand GPCR signalling, functions and pharmacology (Olsen and English, 2023) (Box 1).

Chimeric mammalian G proteins with C-terminal tails derived from different Gα subunits (Conklin et al., 1993) enable measurement of G protein coupling, such as Gα_i/o_ coupling (Coward et al., 1998). For example, a chimeric Gα_q_ protein (Gqi5) containing the last five residues of Gα_i_ enables Gα_i_-coupled receptors to couple to Gα_q_ pathways and generate Ca^2+^ signals that can be measured using fluorescence imaging plate reader assays (Coward et al., 1999). However, measurements of Gα_s_ coupling are problematic with this type of platform, with a Gα_s_ chimera (Gqs5) showing no activity in response to stimulation of the β2-adrenoceptor (also known as ADRB2) (Conklin et al., 1996). The transforming growth factor-α (TGF-α) shedding assay is another chimeric G protein platform based on Gα_q_ that has been used to characterise the signalling profiles of many receptors (Inoue et al., 2012, 2019a). An alternative chimeric strategy leverages yeast pheromone signalling, which involves a single GPCR–G protein interaction between the pheromone receptor Ste2 and the G protein Gpa1. By replacing endogenous Gpa1 with a chimera in which the last five residues are derived from a mammalian G protein and replacing Ste2 with a mammalian GPCR, signalling (assayed via, for example, reporter activation or growth) occurs only when the mammalian GPCR couples to the chimeric G protein (Brown et al., 2003). This enables the G protein-coupling profile of a given GPCR to be determined (Bertheleme et al., 2013; Weston et al., 2014; Shaw et al., 2019).

Initially, individual G protein couplings were determined by measuring binding of GTPγS, a non-hydrolysable or slowly hydrolysable GTP analogue, upon agonist stimulation of GPCRs (Hilf et al., 1989). Now, luminescence-based techniques are commonly used to evaluate G protein activation by measuring dissociation of the heterotrimeric complex, based on either decreased luminescence from a split luciferase or a decrease in BRET. The TRUPATH biosensors measure receptor-catalysed G protein dissociation or rearrangement via BRET (Olsen et al., 2020), using an array of different plasmid-encoded Gα–RLuc8, Gβ and Gγ–GFP2 subunits (Box 1). Shortcomings of this approach include the requirement for simultaneous transfection of multiple plasmids encoding tagged G proteins, where co-transfection efficiencies can be low. Variable expression of individual components can alter the baseline BRET signal, which is also influenced by the constitutive activity of the receptor, thereby making estimations of constitutive receptor activity difficult. To address some of these issues, tricistronic G protein (G-CASE) BRET sensors encoding all three tagged G protein subunits on a single plasmid (Schihada et al., 2021) offer simplified transfection and increased sensitivity for measuring constitutive G protein activity. G protein activity can also be measured using a split NLuc system (NanoLuc binary technology, or NanoBiT), using Gα tagged with a large NanoBiT subunit (LgBiT) and Gβ or Gγ tagged with a small NanoBiT subunit (SmBiT), which interfere minimally with G protein activity (Inoue et al., 2019a), but this system has relatively low light output. Notably, for each of these biosensors there is the potential for the sensor to alter G protein coupling due to its modifications (Wright et al., 2024).

BRET sensors have also been developed to detect activation of endogenous G proteins by unmodified GPCRs (Box 1). In the G protein effector membrane translocation assay (EMTA), upon G protein activation, *Renilla* luciferase II (RlucII)-tagged effector proteins are recruited to the membrane, where they undergo BRET with *Renilla* green fluorescent protein (rGFP) tethered to the membrane via a CAAX motif (Avet et al., 2022). These effectors are selective for different G protein families [p63-RhoGEF (ARHGEF25), Rap1GAP or PDZ-RhoGEF (ARHGEF11) for Gα_q/11_, Gα_i/o_ or Gα_12/13_, respectively] but cannot distinguish between individual members within the same family (Avet et al., 2022). Endogenous Gα_s_ signalling cannot currently be detected with this method due to the lack of selective effectors downstream of Gα_s_. Activation of Gα_s_ protein can instead be measured via BRET between RlucII-tagged Gα_s_ protein and rGFP–CAAX. By measuring colocalisation between Gα subunits and location-specific biosensors (e.g. sensors confined to endosomes), G protein activation can be measured across different cellular compartments and any location bias can be determined (Eiger et al., 2022).

The BRET sensor with ER/K linker and YFP (BERKY) system detects Gα-GTP upon activation of GPCRs (Janicot et al., 2023; Maziarz et al., 2020). In this system, a membrane-anchored NLuc is attached to YFP and linked to a G protein family-specific detector by an ER/K α-helical linker. GPCR activation releases Gα-GTP, allowing it to bind to the detector, which brings YFP close to the NLuc, increasing BRET. The BERKY system has been used for Gα_i_, Gα_q_, Gα_13_ and Rho GTPases (Janicot et al., 2023^;^ Maziarz et al., 2020). A similar system using the C terminus of G protein-coupled receptor kinase 3 (GRK3ct) as the detector can measure free Gβγ (Hollins et al., 2009). In these approaches, transfection of the sensor is required, but not overexpression of G protein. However, BERKY systems are currently unable to assess responses for all heterotrimeric G proteins (especially Gα_s_) and do not have selectivity for individual G proteins within the same family.

More recently, the same detector sequences used in the BERKY system have been used in the development of the ONE-GO biosensors for studying GPCR activation (Janicot et al., 2024), with the addition of a sequence capable of detecting Gα_s_-GTP. This system uses a YFP-tagged Gα subunit, which when bound to GTP has a higher affinity for the NLuc-tagged detector molecule, thus increasing BRET. Unlike the EMTA platform, these detector molecules are reported to not interfere with G protein signalling (Maziarz et al., 2020), and the use of endogenous Gβγ subunits circumvents the need for optimal pairings. Furthermore, these constructs are available on a single plasmid and exhibit a dynamic range suitable for the measurement of endogenous signalling in primary cells.

## Assessing cAMP signalling

Production of cAMP by adenylyl cyclases is modulated by both Gα_s_ and Gα_i/o_ proteins (Fig. 1). Competitive immunoassays utilising homogenous time-resolved FRET (HTRF) can measure changes in intracellular cAMP concentrations with high sensitivity and throughput (Clark et al., 2021; Pearce et al., 2022; Preti et al., 2022) (Box 2). In these assays, cAMP in cell lysates competes with a labelled cAMP analogue for binding to a fluorescent anti-cAMP antibody. Using long-lived lanthanide cryptate or lanthanide chelate donors creates a delay between excitation and detection and reduces background noise, improving sensitivity. Alternatively, genetically encoded luminescent or fluorescent sensors can measure cAMP dynamics in live cells, and these can be targeted to specific subcellular compartments if required. Many of the available cAMP sensors (Greenwald et al., 2018) were developed using fragments of protein kinase A (PKA) or exchange protein activated by cAMP [EPAC; herein referring to both EPAC1 (RAPGEF3) and EPAC2 (RAPGEF4)]. Examples include the cADDis cAMP indicators (Tewson et al., 2018), which are composed of the cAMP-binding domain of EPAC fused to a variety of circularly permuted fluorescent proteins (i.e. fluorescent proteins in which the original N and C termini are fused via a linker sequence, thereby creating new termini near the chromophore). This circular permutation allows conformational changes of sensor domains fused to the new N and C termini to affect the nearby chromophore and alter fluorescence. These sensors can be packaged in baculovirus to enable expression in primary cells (Tewson et al., 2018). Green fluorescent cAMP indicator (G-FLAMP) (Wang et al., 2022) and variants such as G-FLAMP2 (Liu et al., 2022) employ a bacterial cyclic nucleotide-binding domain (CNBD) fused to a circularly permuted GFP (Box 2). These bacterial CNBD-based sensors mitigate concerns that PKA- and EPAC-based sensors may influence mammalian signalling pathways.

The optimal choice of assay depends on factors including the duration and magnitude of the cAMP responses being measured. Longer-duration time-lapse experiments may be suited to BRET or FRET sensors, such as cAMP sensor using YFP–EPAC–RLuc (CAMYEL) (Jiang et al., 2007) and cAMP universal tag for imaging experiments (CUTie) (Surdo et al., 2017), or fluorescence intensity sensors like G-FLAMP (Liu et al., 2022) (Box 2). EPAC-based cAMP sensors can be targeted to specific cellular compartments, such as the cytoplasm (Nikolaev et al., 2004) or nucleus (Wachten et al., 2010), to provide spatial information. For measuring lower-magnitude cAMP responses, bioluminescent complementation sensors such as pGloSensor-22f can achieve high sensitivity and dynamic range, with up to ∼1000-fold changes in luminescence (Wang et al., 2022). Alternatively, small reductions in cAMP signalling associated with Gα_i/o_ activation can require the sensitivity of HTRF (Preti et al., 2022). When imaging single-cell cAMP responses, fluorescent G-FLAMP variants are likely of greatest utility due to their greater brightness.

## Measuring Ca^2+^ signalling responses

Activation of Gα_q/11_-coupled GPCRs stimulates phospholipase C (PLC), producing inositol 1,4,5-trisphosphate (IP_3_) from phosphatidylinositol 4,5-bisphosphate (PIP_2_) at the plasma membrane. IP_3_ then activates IP_3_ receptors in the endoplasmic reticulum (ER) membrane, which release ER luminal Ca^2+^ to generate cytosolic Ca^2+^ signals (Berridge et al., 1999) (Fig. 1). Gα_i/o_ signalling also influences this pathway via interactions between Gβγ and PLC (Falzone and MacKinnon, 2023; Pfeil et al., 2020). Gα_s_-derived Gβγ is also reported to drive PLC activation and Ca^2+^ signalling (Brands et al., 2024). Activation of this pathway can be detected using an HTRF-based kit that uses an antibody specific to inositol monophosphate, a downstream metabolite of IP_3_, as a proxy for Gα_q_ activation (Johnstone et al., 2022). Direct measurement of cytosolic Ca^2+^ can be achieved using Ca^2+^-sensitive dyes (Lock et al., 2015) or protein-based Ca^2+^ sensors (Heim and Griesbeck, 2004; Li et al., 2023a,b; Zhang et al., 2023) (Box 2).

Ca^2+^_­_-sensitive dyes are often conjugated to acetoxymethyl (AM) ester groups to confer membrane permeability and allow trapping of the active Ca^2+^ indicator inside cells after cleavage by cellular esterases. Single-wavelength Ca^2+^ indicators include Fluo-4, Oregon Green BAPTA-1, Cal-520 and the Calbryte series, whereas dual-wavelength (ratiometric) indicators include Fura-2 and Indo-1 (Lock et al., 2015; Paredes et al., 2008). Ratiometric indicators control for variations in dye concentration and can facilitate calibration to measure absolute concentrations of Ca^2+^. Ca^2+^ binding affinities differ widely between dye-based indicators [with dissociation constant (*K*_d_) values in the nanomolar to millimolar range], enabling compartment-specific measurements wherein high-affinity and low-affinity indicators can show changes in fluorescence at low concentrations of Ca^2+^ (e.g. in the cytosol) and high concentrations of Ca^2+^ (e.g. within the ER), respectively (Paredes et al., 2008). The emission spectra of indicators span the visible range from blue to far red, with green-emitting Ca^2+^ dyes often being the brightest (Lock et al., 2015).

There are two main classes of genetically encoded Ca^2+^ sensors: cameleon and GCaMP (Heim and Griesbeck, 2004; Miyawaki et al., 1997). The cameleon type relies on FRET between fluorescent donor and acceptor proteins linked to calmodulin (CaM) and a Ca^2+^–CaM-binding peptide, respectively, within a single fusion protein. The GCaMP type is based on a single circularly permuted fluorescent protein fused at its termini to CaM and a Ca^2+^–CaM-binding peptide. Ca^2+^ binding leads to changes in the environment of the chromophore, which alters the fluorescence signal (Li et al., 2023a,b; Zhang et al., 2023). GCaMP-type sensors generally exhibit larger fractional changes in fluorescence and are available in a wider range of colours, whereas cameleon-type sensors are ratiometric. Of the existing GCaMP-type sensors, the ‘NEMO’ Ca^2+^ sensors are proposed to be best in class in terms of dynamic range and brightness (Li et al., 2023a), and the jGCaMP8 sensors best in class in terms of kinetics (Zhang et al., 2023). GCaMP sensors of different colours exist (Zhang and Looger, 2024), for example, in the ranges of ultraviolet (Zhao et al., 2011), blue (Inoue et al., 2019b; Zhao et al., 2011), green (Chen et al., 2013; Li et al., 2023a,b; Zhang et al., 2023), yellow (Mohr et al., 2020), red (Dana et al., 2016; Inoue et al., 2019b; Shen et al., 2018), far red (Subach et al., 2019) and near-infrared (Qian et al., 2019).

Other types of Ca^2+^ sensors also exist. The CaMPARI Ca^2+^ sensors are photoconvertible fluorescent proteins, which in high Ca^2+^ conditions convert irreversibly from green to red upon illumination with violet light, providing a ‘memory’ of changes in cellular Ca^2+^ concentration (Das et al., 2023, 2022). Chemigenetic Ca^2+^ indicators, which utilise interactions between fluorescent dyes and protein quenchers, offer improved brightness and spectral adaptability (Farrants et al., 2024). Existing luminescent Ca^2+^ indicators have relatively high brightness and contrast, with Ca^2+^ affinities in the physiological range (Tian et al., 2022; Lambert et al., 2023 preprint). For example, the CaBLAM indicators incorporate a shrimp luciferase variant termed sensor scaffold luciferase (SSLuc) with an inserted sensor domain that makes it sensitive to Ca^2+^ concentrations (Lambert et al., 2023 preprint).

An advantage of these genetically encoded Ca^2+^ sensors is that they can be targeted to subcellular compartments to measure local Ca^2+^ signals (Suzuki et al., 2014). In the future, optimised far red and near-infrared Ca^2+^ sensors might help to minimise autofluorescence while also maximising tissue penetration and providing opportunities for multicolour imaging with other indicators.

## Intracellular protein mediators of G protein signalling

The activity of numerous intracellular proteins, such as kinases and small monomeric G proteins, is dependent on GPCRs. The pleiotropy of G protein coupling, crosstalk between pathways and cell-type specific responses generates a need for a wide variety of sensors to measure multiple responses simultaneously. For example, the sensitive and multimodal protein-based sensors exRai-AKAR2 and exRai-CKAR report on substrate phosphorylation of PKA and protein kinase C (PKC), respectively (Mehta et al., 2018; Zhang et al., 2021). The phosphorylation of these substrates is indicated by increased fluorescence intensity from a single fluorophore, which can be used to detect kinase activity in single cells or in high-throughput plate-based assays, by flow cytometry or *in vivo* (Mehta et al., 2018; Zhang et al., 2021). Furthermore, tethering exRai-AKAR2 (Zhang et al., 2021) to subcellular compartments has demonstrated the importance of endosomal β2-adrenoceptor signalling for the initiation of transcriptional events in the nucleus (Willette et al., 2023). Unlike the PKA and PKC sensors, RAB-EKARev, a sensor for activity of extracellular signal-regulated kinase (ERK; herein referring to ERK1 and ERK2, also known as MAPK3 and MAPK1, respectively), uses a complementation approach to increase fluorescence upon phosphorylation (Mehta et al., 2018). The FRET- and BRET-based reporters EKAR (Harvey et al., 2008; Eiger et al., 2022) and REV (Xu et al., 2013) are also based on an ERK phosphorylation targeting sequence, and both have the capacity for nuclear and cytoplasmic targeting, providing spatial resolution for measuring signalling. For primary cells or other cells that are difficult to genetically modify, commercial kits are available that can detect specific phosphorylation of endogenous ERK or its targets (Cecon et al., 2021; Guo et al., 2024).

Monomeric G proteins such as RhoA can be activated by GPCR signalling through various RhoGEF proteins. RhoA acts downstream of Gα_12/13_, but biphasic RhoA activation, which is dependent on two different RhoGEFs, might also follow Gα_q/11_-mediated responses, as observed using a FRET-based RhoA sensor (DORA-RhoA) (van Unen et al., 2015). A BRET-based version of DORA-RhoA, utilising NLuc and Venus, has revealed Gα_12/13_ coupling to the thromboxane A2 receptor (Masuho et al., 2023).

## β-arrestins and GRKs

**Fig. 3.**
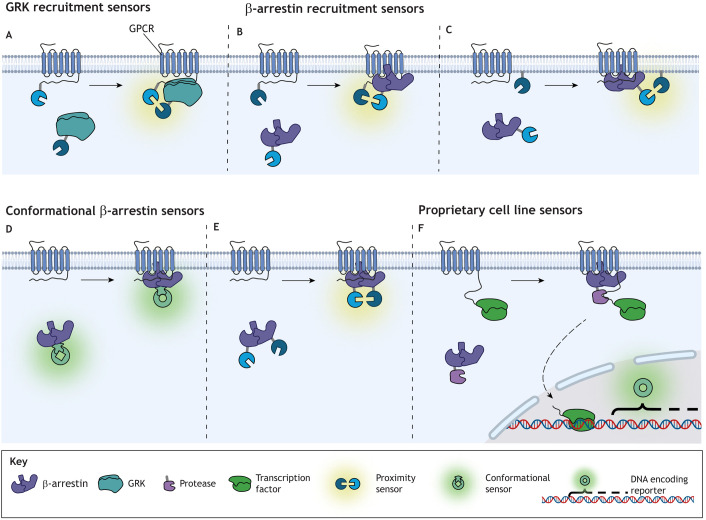
**Measuring β-arrestin and GPCR kinase recruitment.** (A) Biosensors to measure GRK activity during the initial steps in GPCR desensitisation are relatively limited and rely on RET between a tagged GPCR and GRK. (B–D) Biosensors to measure β-arrestin recruitment are diverse and can be broadly classified into three types: proximity based, conformational change based and transcriptional reporter based. Proximity-based sensors are commonly used to measure proximity of the recruited β-arrestin to (B) a GPCR or (C) the plasma membrane. Other sensors measure recruitment of β-arrestin by detecting a conformational change via either (D) increased signal from a fluorescent conformational sensor fused to β-arrestin, or (E) an intramolecular proximity sensor. (F) Proprietary assays use cell lines expressing a GPCR fused to a transcription factor and a β-arrestin tagged with a protease, which cleaves the transcription factor from the intracellular C terminus of the receptor when recruited, increasing the synthesis of a reporter gene. Created in BioRender by Redfern-Nichols, T., 2025. https://BioRender.com/z75j601. This figure was sublicensed under CC-BY 4.0 terms.

Following activation, the functional responses of GPCRs can decrease in a process termed desensitisation, despite the continuous presence of a stimulus. This process can occur via a variety of mechanisms including phosphorylation, recruitment of β-arrestins, receptor internalisation and degradation. GRKs can phosphorylate GPCRs after activation, facilitating recruitment of β-arrestin 1 and β-arrestin 2 (Yin et al., 2009) and receptor internalisation (Gurevich and Gurevich, 2019), causing desensitisation (Fig. 1). A BRET-based GRK sensor consisting of a Venus-tagged GRK and a luciferase-tagged GPCR has been used to report recruitment of GRKs to GPCRs following receptor activation (Gardner et al., 2024) (Fig. 3A). Perturbation of GRK expression can alter GPCR desensitisation or β-arrestin recruitment (Møller et al., 2020; Pearce et al., 2022) and reveal physiological roles of downstream signalling. β-arrestin assays detect translocation of β-arrestin to the plasma membrane and the GPCR (Fig. 3B,C) or conformational changes of β-arrestin following receptor binding. The conformational assays utilise changes in fluorescent protein conformation (Brouwers et al., 2021) (Fig. 3D) or BRET between two distinct parts of the β-arrestin (Lee et al., 2016; Oishi et al., 2020) upon recruitment to a receptor (Fig. 3E); both strategies utilise untagged GPCRs. The Borealis arrestin biosensor (Montana Molecular) also relies on a conformational change in the β-arrestin, with recruitment of the biosensor to the GPCR reducing fluorescence intensity (Hoare et al., 2020). This biosensor is packaged within a BacMam vector (to enable baculovirus-mediated expression in mammalian cells) alongside the receptor of choice; therefore, target versatility is determined by availability from the supplier. MULTISCREEN, a proximity-based β-arrestin assay, uses a split luciferase to measure translocation of β-arrestins to the plasma membrane; intact luciferase is formed when a tagged β-arrestin and a membrane anchor protein, GAP43, are in proximity (Dogra et al., 2016). This approach is akin to how β-arrestin activity is measured via the EMTA platform, with translocation of RlucII–β-arrestin to the membrane resulting in increased BRET with rGFP–CAAX. An alternative proximity-based assay measures BRET between a luciferase-tagged GPCR and a YFP-tagged β-arrestin (Marti-Solano et al., 2020; Pearce et al., 2022). β-arrestin recruitment can also be measured using NanoBiT, with the β-arrestin and GPCR tagged with LgBiT and SmBiT, respectively (Littmann et al., 2019). Additional proximity-based assays comprise proprietary cell lines engineered to express tagged GPCR–β-arrestin combinations. For example, the Tango GPCR assay (Thermo Fisher Scientific) uses a protease-tagged β-arrestin to cleave a transcription factor from a GPCR–transcription factor fusion protein, leading to expression of β-lactamase, the levels of which can be detected through substrate administration (Dogra et al., 2016). The PathHunter assay (DiscoverX) uses a split β-galactosidase attached to the GPCR and β-arrestin, with complementation detected through substrate hydrolysis (Yin et al., 2009) (Fig. 3F). An HTRF-based kit (Revvity) can detect the recruitment of untagged β-arrestins using antibodies against β-arrestin 2 and clathrin adaptor protein 2 (AP2) complex (Bous et al., 2022; Liu et al., 2024 preprint; Wang et al., 2023). This assay enables detection in primary cells but relies on recruitment of the AP2 complex to β-arrestin 2 – which does not always occur – and due to heterologous GPCR desensitisation (i.e. activation of one GPCR leading to desensitisation of other GPCRs that have not been stimulated directly by agonist), it is likely not receptor specific.

## Quantifying internalisation and subcellular trafficking of GPCRs

**Fig. 4.**
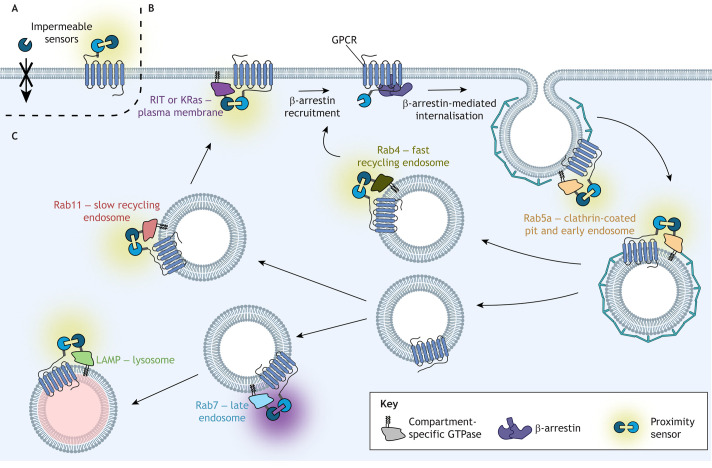
**Proximity sensors for studying GPCR internalisation and subcellular trafficking.** Internalisation can be measured by assessing the loss of a GPCR from the plasma membrane, either from (A) a reduction in the interaction between a tag on the extracellular N terminus of the GPCR and a membrane-impermeable component in the extracellular medium, or (B) a bystander interaction with a plasma membrane-associated GTPase (such as RIT or KRas). (C) Similar bystander interactions can be applied to measure the interaction of internalised GPCRs with monomeric GTPases specific to different subcellular compartments. These GTPases include Rab5a at clathrin-coated pits or early endosomes, Rab4 at fast recycling endosomes, Rab11 at slow recycling endosomes and Rab7 at late endosomes. A lysosomal marker, lysosome-associated membrane glycoprotein (LAMP, also known as LAMP1), is also used to indicate receptor degradation. Created in BioRender by Redfern-Nichols, T., 2025. https://BioRender.com/e65u934. This figure was sublicensed under CC-BY 4.0 terms.

Following activation, GPCRs undergo internalisation to intracellular membrane compartments, where they can continue signalling, be degraded or be recycled back to the membrane (Fig. 4). The rate and extent of internalisation, as well as subcellular trafficking, are important for determining agonist efficacy and cellular responses (Calebiro and Godbole, 2018). GPCR internalisation can be monitored via fluorophore-tagged receptors or by using antibodies, enabling fluorescence microscopy or enzyme-linked immunosorbent assays (ELISAs). However, these experiments are time consuming and relatively low throughput, and acquiring kinetic data can be difficult. Alternatively, loss of GPCR expression at the cell surface can be measured in real time using a cell-impermeable component, producing a loss of signal upon receptor internalisation (Fig. 4A). For example, the HiBit extracellular detection kit (Promega) is based on a split luciferase, with one part of the reporter present only in the extracellular medium (Boursier et al., 2020; Rouault et al., 2017). The diffusion-enhanced resonance energy transfer (DERET) internalisation assay uses a SNAP-tagged GPCR labelled with a cell-impermeable substrate. FRET between the SNAP-labelled GPCR and the acceptor in the extracellular medium is only possible when the GPCR is in the plasma membrane (Levoye et al., 2015). Alternatively, SNAP-tagged GPCRs fluorescently labelled on their extracellular domains using cleavable probes can be used to monitor internalisation by measuring the resistance to reduction and cleavage by a membrane-impermeant reducing agent such as sodium 2-mercaptoethanesulfonic acid (MESNA) (Hinds et al., 2023); a greater resistance to cleavage indicates increased internalisation of the GPCR.

Proximity-based assays can also assess the loss of ‘bystander’ interactions between GPCRs and plasma membrane markers, such as the membrane-bound GTPases KRas and RIT (also known as RIT1) (Lan et al., 2011; Pearce et al., 2022) (Fig. 4B). Moreover, subcellular trafficking of GPCRs can be monitored and quantified using proximity markers for different endosomal compartments, such as Rab5a, which is localised to early endosomes (Harris et al., 2021 preprint; McNeill et al., 2024; Pearce et al., 2022). Some of the most commonly used markers for different compartments in the endocytic pathway are shown in Fig. 4C, although many others exist.

## Transcriptional events

GPCR activation can lead to transcriptional changes mediated by the action of transcription factors on genetic response elements (GREs) (Hill et al., 2001). For example, activation of Gα_s_ leads to phosphorylation of cAMP-responsive element-binding (CREB) nuclear transcription factors by PKA. Phosphorylated CREB proteins then bind to genomic cAMP response elements (CREs) to alter transcription. When encoded downstream of GREs, reporters such as luciferases can provide useful tools for high-throughput drug screening (Zhang and Xie, 2012). Addition of hPEST (Pro-Glu-Ser-Thr) sequences, which act as degradation signals, destabilises the luciferase reporter, providing greater fold induction by reducing basal luciferase concentration (Cheng et al., 2010). Other GREs include the nuclear factor of activated T-cells response element (NFAT-RE), serum response element (SRE), serum response factor response element (SRF-RE), nuclear factor-kappa B (NF-κB), and signal transducer and activator of transcription response elements (STAT-REs). These GREs can be regulated by a wide variety of potential upstream activators in different cell types (Cheng et al., 2010; Gao et al., 2004; Pelletier et al., 2003). For example, NFAT-REs are targeted downstream of PKC activation. Strategies allowing for multiplexing of orthogonal luciferase reporters, such as the use of luciferase-specific substrates and spectral deconvolution, can enable the measurement of multiple GRE activities simultaneously (Sarrion-Perdigones et al., 2019).

## Label-free approaches

Overexpressing tagged signalling proteins in cells as part of biosensor assays risks perturbing the innate G protein coupling and signalling of GPCRs (Box 1). It has been shown that different G protein biosensor platforms detect different G protein couplings from a given receptor (Wright et al., 2024) and that increased expression of a GPCR can remove the signalling bias that is observed when the GPCR is expressed at a lower, endogenous level (Li et al., 2023b). Commercially available kits to measure accumulation of second messengers partially overcome this but are costly and often do not provide kinetic, live-cell readouts. For this reason, whole-cell, label-free assays have been developed to measure the kinetics of GPCR signalling in live cells. The xCELLigence real-time cell analyser system (Agilent) can be used to measure GPCR signalling based on electrical impedance generated by cells plated on microelectronic plates, which depends on factors such as confluency, adhesion, viability and morphology – all of which can be influenced by GPCR signalling (Doornbos and Heitman, 2019). A second label-free methodology is dynamic mass redistribution (DMR), which detects changes in refractive index near a biosensor induced by GPCR signalling that can cause a redistribution of cellular proteins, cytoskeletal reorganisation or a detectable redistribution of mass (Seibel-Ehlert et al., 2021). Both assays represent a more holistic approach to studying GPCR signalling and can be used in relatively unperturbed, live cells; however, these assays do not offer definition of the downstream signalling components involved in generating responses.

## Conclusions

In this Review, we have presented a critical overview of the state-of-the-art approaches for investigating GPCR activation and downstream signalling pathways, to facilitate mechanistic insight and expand the understanding of GPCR function in cells. In the past 25 years, measurements of GPCR signalling have advanced from low-throughput assays such as [^35^S]GTPγS binding for G protein coupling (Harrison and Traynor, 2003) and co-immunoprecipitation for β-arrestin recruitment (Luttrell et al., 1999) to a variety of quantitative assays that can measure GPCR activation and downstream signalling in real time, in live cells. These advances have led to rapid progress in our understanding of GPCR functions and the development of therapeutics. Challenges remain, such as developing strategies to measure GPCR signalling quantitatively whilst minimising the perturbative effects of the assay employed. Overexpressed and tagged biosensors can generate robust real-time signals in live cells to identify specific signalling events and protein–protein interactions, but they can also perturb the very signalling cascades they attempt to measure. Label-free approaches and the use of HTRF kits can enable the detection of endogenous GPCR signalling in cells. However, label-free approaches often lack the ability to define the specific signalling components involved, whereas HTRF kits often require cell lysis and can be lacking in temporal information. Therefore, combining the information derived from different assays may provide the most complete, quantitative understanding of GPCR signalling. Further progress in the development of new methods will enable an enhanced understanding of GPCR functions, cell signalling and pharmacology.
